# Management and modeling approaches for controlling raccoon rabies: The road to elimination

**DOI:** 10.1371/journal.pntd.0005249

**Published:** 2017-03-16

**Authors:** Stacey A. Elmore, Richard B. Chipman, Dennis Slate, Kathryn P. Huyvaert, Kurt C. VerCauteren, Amy T. Gilbert

**Affiliations:** 1 United States Department of Agriculture, National Wildlife Research Center, Fort Collins, Colorado, United States of America; 2 United States Department of Agriculture, Wildlife Services, National Rabies Management Program, Concord, New Hampshire, United States of America; 3 Department of Fish, Wildlife, and Conservation Biology, Colorado State University, Fort Collins, Colorado, United States of America; Swiss Tropical and Public Health Institute, SWITZERLAND

## Abstract

Rabies is an ancient viral disease that significantly impacts human and animal health throughout the world. In the developing parts of the world, dog bites represent the highest risk of rabies infection to people, livestock, and other animals. However, in North America, where several rabies virus variants currently circulate in wildlife, human contact with the raccoon rabies variant leads to the highest per capita population administration of post-exposure prophylaxis (PEP) annually. Previous rabies variant elimination in raccoons (Canada), foxes (Europe), and dogs and coyotes (United States) demonstrates that elimination of the raccoon variant from the eastern US is feasible, given an understanding of rabies control costs and benefits and the availability of proper tools. Also critical is a cooperatively produced strategic plan that emphasizes collaborative rabies management among agencies and organizations at the landscape scale. Common management strategies, alone or as part of an integrated approach, include the following: oral rabies vaccination (ORV), trap-vaccinate-release (TVR), and local population reduction. As a complement, mathematical and statistical modeling approaches can guide intervention planning, such as through contact networks, circuit theory, individual-based modeling, and others, which can be used to better understand and predict rabies dynamics through simulated interactions among the host, virus, environment, and control strategy. Strategies derived from this ecological lens can then be optimized to produce a management plan that balances the ecological needs and program financial resources. This paper discusses the management and modeling strategies that are currently used, or have been used in the past, and provides a platform of options for consideration while developing raccoon rabies virus elimination strategies in the US.

## Introduction

Rabies is an ancient viral disease that is still a global concern in both humans and animals [1, 2]. Rabies virus (RABV) is transmitted primarily through bite contact with reservoir species, and the disease is usually fatal once clinical signs appear [1]. An estimated 59,000 people die of rabies every year, mostly due to bites from domestic dogs (*Canis familiaris*) in developing parts of the world [3, 4]. In the US and Canada, wildlife species are the primary reservoirs of rabies because of successful control and elimination of rabies in dogs through widespread availability and use of parenteral vaccines, restricted animal movements, as well as public awareness and responsible pet ownership [5, 6, 7]. In relation to other wildlife diseases, rabies maintains a relatively high profile because it is zoonotic, has one of the highest case fatality rates of any infectious disease, and because of ongoing management of the disease in reservoir populations. In a broader public health perspective, however, rabies remains neglected and subject to political will and available resources, which fluctuate in response to disease burden and surveillance activities [8, 9].

In the US, about 34,000 courses of post-exposure prophylaxis (PEP) are administered per annum [10]; the per capita rate of PEP administration is almost twice as high in the states where the raccoon (*Procyon lotor*) variant of RABV circulates than in states with skunk variants and three and six times as high compared to where arctic fox and bat variants circulate, respectively [10]. Furthermore, an average of 6,000 cases of animal rabies are reported in the US each year, and the majority of these cases are diagnosed from the area where the raccoon variant of RABV is enzootic, a trend that is partly associated with elevated rates of spillover of raccoon variants into other animals [11]. Beyond the public health risk, the circulation of RABV in wildlife results in other human–wildlife conflicts, such as spillover transmission to livestock [12] or endangered species [13, 14]. Additionally, there is an intrinsic human desire for healthy wildlife populations and concern for the reservoir species impacted by rabies [15]. Although rabies is a vaccine-preventable disease, a rich history of folklore, high case fatality rate, and anxiety surrounding the unknown enables an innate fear of rabies to persist, even among health care workers [16, 17]. This fear might drive unnecessary precaution and lead to an over-administration of PEP, adding to the overall cost of disease mitigation [10, 17, 18].

In response to threats to public health, agriculture, and free-ranging wildlife populations posed by RABV, several methods of rabies control targeting animal populations have been implemented worldwide, including local population reduction, parenteral vaccination, oral vaccination, or combinations of these methods. In this paper, we review the management strategies traditionally used to control rabies in wildlife, highlighting both successes and challenges, with special consideration for application to raccoons. We also discuss the modeling approaches that have been used to better understand rabies ecology in wildlife, with the intention of translating these strategies to enhance raccoon rabies management to achieve elimination in North America.

## Background

**Fig 1:**
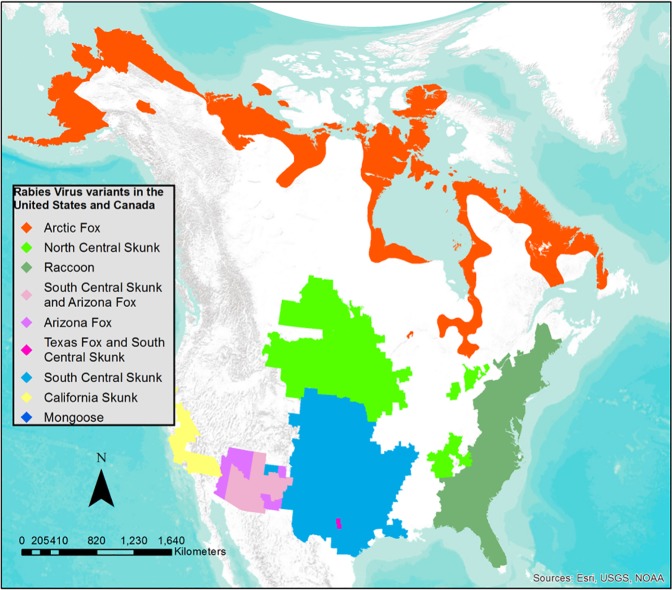
Current geographic distribution of rabies virus variants in the continental US and Canada.

In the continental US, seven distinct RABV variants circulate in four terrestrial wildlife species: raccoons, skunks (principally *Mephitis mephitis*), and foxes (arctic fox: *Vulpes lagopus* and gray fox: *Urocyon cinereoargenteus*). These variants are maintained within specific reservoirs and have recognized geographic distributions (Fig 1) [8]. The RABV variants in the US and Canada evolved from two major lineages: dog RABV (California skunk, north-central skunk, Arizona gray fox, Texas gray fox, and Arctic fox variants) or bat RABV (raccoon and south-central skunk variants) [5]. Raccoon rabies cases have been reported annually in Florida since 1953 [19]. In the 1960s and 1970s, a northward range expansion brought raccoon rabies into neighboring states at a rate of 40 km/year by 1977 [20]. In 1977, a translocation of raccoons from Florida to West Virginia initiated an epizootic event that progressively spread throughout the eastern US and into eastern Canada [21, 20]. The development of monoclonal antibody typing methods in the mid to late 1970s [22] allowed for the identification of RABV variants adapted to specific wildlife hosts. By 1999, the raccoon variant was responsible for the highest number of rabid companion animals reported in the US [23]. While raccoons might become infected with other RABV variants through spillover [11], in this paper all references to raccoon rabies is related to the specific RABV variant that is adapted to and circulates in raccoons in the eastern US [11].

Due to the occurrence of raccoon rabies over a wide geographic range, management efforts are highly collaborative and demand intersectoral cooperation from diverse domestic and international partners and stakeholders. The implementation of the US Department of Agriculture (USDA), Animal and Plant Health Inspection Service (APHIS), Wildlife Services, National Rabies Management Program (NRMP) in 1999, and the signing of the North American Rabies Management Plan (NARMP) in 2008, have helped to provide focus, leadership, and partnerships within and across the US, Canada, and Mexico, thus enhancing the collaborative nature of rabies management [24].

A major focus of NRMP activities and the NARMP is the management and elimination of raccoon rabies from the eastern US and Canada. As demonstrated by campaigns targeting fox rabies in Europe [25], the goal of elimination over large landscapes is attainable. The US and Canada also must consider host species differences (e.g., density and behavior), diverse and fragmented landscapes with extensive rural–suburban interface, and generally ubiquitous raccoon populations. These factors present additional challenges above those encountered in admittedly complex fox rabies control programs in Europe. Common areas of uncertainty and complexity that raccoon rabies management programs must navigate include, but are not limited to, environmental and climatic changes, sustainable long-term funding, spillover of raccoon RABV into sympatric skunk (i.e., *M*. *mephitis*) populations [26, 8], and translocation of reservoir animals [27].

Raccoons seem to thrive in almost any part of the urban–rural gradient, occurring at especially high densities in suburban areas where their tolerance of humans and flexible diet allow them to find food and shelter from both anthropogenic and natural sources [28, 29]. Raccoon densities and home range sizes can vary greatly across habitat types [30, 31, 32]. Details regarding habitat-dependent movement and contact structure of infected raccoons are important to learn, especially for identification of ecological corridors [33] that might be good locations to employ targeted management activities. Due to their peridomestic habits, importance as a rabies reservoir, and the critical nature of host movement to understand disease spread, understanding raccoon movement remains an active area of study [33, 34, 35]. The emergence of network analysis approaches, and technological advances in tracking marked animals including GPS and proximity collars, has offered new insight into raccoon social behavior and ecology [29, 36, 37, 38, 39].

Translocation (both purposeful and involuntary) is commonly associated with raccoons and can jeopardize rabies management efforts if an infected raccoon is introduced to a naïve or previously managed area [21, 27]. Raccoons and other wildlife species that scavenge human refuse can be accidentally transported over potentially large distances by garbage trucks or other vehicles [27]. Also, raccoons are involved in a high volume of wildlife damage or “nuisance” complaints and are likely to be trapped in urban or suburban areas by the public, wildlife rehabilitators, or wildlife control personnel and might subsequently be released elsewhere (legally or illegally), thereby enhancing the potential geographic spread of RABV and other pathogens [28].

The design and success of rabies control strategies is influenced by viral transmission dynamics within the target species population, which is a product of the host and pathogen relationship. Pathogen transmission is often characterized as density-dependent or independent (frequency dependent), but a combination of the two can also occur. The dominance of one transmission type over another might be a scale-dependent question or might be related to whether the current infection dynamics are acute (epizootic) or enzootic [14]. If virus transmission within a target population is not density-dependent or if the movement, foraging, mating, or other pertinent behaviors of host species are not well understood, then disease-control strategies such as local population reduction can be ineffective [14, 40]. However, determining the role of population density in rabies transmission is not trivial, especially for wildlife, for which field data on many behavioral and population processes remain largely unobserved [41]. Also, it is difficult to generalize regarding RABV transmission dynamics because of the ecological diversity of genetic variants, reservoir hosts, and habitats. As such, it is not surprising to find varying and sometimes conflicting descriptions of rabies transmission dynamics in wildlife [14, 41].

## Rabies management strategies

### Oral rabies vaccination

One of the most successful methods of RABV control has been through landscape-scale oral rabies vaccination (ORV) programs. Since 1978, ORV has been used to eliminate the virus from red foxes in western Europe and reduced the disease incidence in central Europe [42]. In Ontario, Canada, aerially distributed Evelyn-Rokitnicki-Abelseth (ERA) vaccine baits eliminated an arctic RABV variant from red foxes during the 1990s, although persistence of this variant in skunks led to additional baiting with a then-novel ORV product, ONRAB (Ontario Rabies Vaccine Bait, Artemis Technologies, Guelph, Ontario, Canada) [43]. ORV programs, in conjunction with parenteral vaccination of domestic dogs in the US have been successful in controlling canine rabies in coyotes (*Canis latrans*) in south Texas, leading to the declaration of the US as canine rabies–free in 2007 [44, 45, 46]. Currently, the primary focus of ORV occurs in the eastern US, where coordinated raccoon rabies management programs led by the USDA, APHIS, Wildlife Services and a coalition of other federal, state, county, and municipal agencies, non-governmental organizations (NGOs), and universities are in place to control and eliminate raccoon rabies [10, 47].

Attenuated derivatives of the Street-Alabama-Dufferin (SAD) strain of RABV were used in a variety of baits to eliminate fox rabies from western Europe [25, 48]. In the US, however, only Raboral V-RG (V-RG; Merial Inc., Athens, GA, US), a live recombinant vaccinia virus–vectored vaccine, was available for use from the mid-1990s until 2011, when the experimental use of ONRAB, a live recombinant human adenovirus–vectored vaccine, began. ONRAB is licensed for use in Canada [49], but is still under experimental use in the US, although field trials are nearing completion [50]. The results, in combination with other data requirements, will be used to aid evaluation for licensure in the US. The raccoon variant ORV zone in the eastern US is maintained principally by aerial distribution of baits from fixed-wing aircraft (89% of total baiting activities), although hand-baiting (5%), targeted distribution by helicopter (5%), and bait stations (1%) are also used in some suburban and urban areas [24]. In two comparison studies with V-RG along the US/Canada border, the proportion of antibody-positive raccoons was higher in the ONRAB-baited areas [51, 52]. These data suggested that ONRAB could be a useful addition to ORV programs in the US [51] and served as one underlying basis to begin the US field trials.

Bait distribution (over space and time) and uptake are key variables impacting vaccine responses in target animal populations and vary with ecosystem characteristics and management goals (Table 1). The density of free-roaming target and non-target animals is important for determining the number of baits that should be distributed within a control area [53, 54, 55]. Management of raccoon rabies through ORV requires a higher bait density than may be typically used in programs targeting canids, which are territorial, have larger home ranges, and are less densely populated than raccoons and skunks. For example, during European ORV efforts to eliminate rabies in red foxes, 20–25 baits/km^2^ were commonly distributed [42], whereas bait densities of 75–300 baits/km^2^ have been used to target raccoon and skunk reservoirs in eastern North America (see Table 1).

**Table 1:** Previously published ORV campaigns in North America and the estimated population seroprevalence in response to vaccination.

Species Tested	Study Location	Bait Type	Flight-Line spacing (km)	Bait Density (baits/km^2^)	Post-Bait Seroprevalence (%)	Diagnostic Test Used	Reference
Red foxes	Eastern Ontario, Canada	ERA	1.0–2.0	20	nd	n/a	[150, 49]
Red foxes	Toronto, Ontario, Canada	ERA	n/a	49–69^1^	46–80 (mean = 61)	VNT, ELISA	[65, 151]
Red foxes	Ontario, Canada	V-RG	0.75–1.5	75, 150	7–28 (mean = 14)	cELISA	[152]
Gray foxes	West-central Texas, US	V-RG	0.8	27–39	37–84 (mean = 62)	VNT	[44]
Coyotes	South Texas, US	V-RG	0.8	19–27	18–87 (mean = 56)	VNT	[153, 44]
Raccoons	Anne Arundel County, Maryland, US	V-RG	0.5	75, 100	21–47 (mean = 33)	VNT	[154]
Raccoons	Massachusetts, US	V-RG	n/a	103 (uniform), 93 (targeted), 135(targeted)	16–55 (uniform), 39–67 (targeted), 46–77 (targeted)	VNT	[155]
Raccoons	New Jersey, US	V-RG	n/a	64 (targeted)	7–71 (mean = 41)	VNT	[156]
Raccoons	Wolfe Island, Ontario, Canada	V-RG	1.5	75, 150	nd^2^	n/a	[69]
Raccoons	Parramore Island, Virginia, US	V-RG	n/a	1000	52	VNT	[61]
Raccoons	Ohio, US	V-RG	0.5	75, 150, 300	22, 18, 11^3^, 27, 14, 8^3^, 41, 36, 25^3^	VNT	[157]
Raccoons	Maine, US	V-RG	1.0	75	30–33, 25	VNT, cELISA	[51]
Raccoons	Vermont, US	V-RG	0.75	150	38, 38	VNT, cELISA	[52]
Raccoons	Quebec, Canada	ONRAB	0.75	150	52, 51	VNT, cELISA	[52]
Raccoons	West Virginia, US	ONRAB	0.75	75	49	VNT	[50]
Raccoons	New Brunswick, Canada	ONRAB	1.0	75	75–78, 67	VNT, cELISA	[51]
Skunks	Maine, US	V-RG	1.0	75	3–11, 3	VNT, cELISA	[51]
Skunks	Ontario, Canada	ONRAB	0.25, 0.50	300	20–34^4^, 36–62^5^	cELISA	[43]
Skunks	West Virginia, US	ONRAB	0.75	75	7	VNT	[50]
Skunks	New Brunswick, Canada	ONRAB	1.0	75	15–18, 15	VNT, cELISA	[51]

^1^Straight line along a ravine (unit is baits/km).

^2^Not determined, but apparent elimination of raccoon rabies from the island; used in conjunction with TVR and population reduction.

^3^Based on titers (≥5, ≥12, ≥56, respectively).

^4^Including only strong positives (inhibition value ≥26%).

^5^Including suspect and strong positives (inhibition value ≥16%).

VNT, virus neutralization test; ELISA, enzyme-linked immunosorbent assay; cELISA, competitive ELISA; nd, not determined; n/a, not applicable.

Landscape heterogeneity and season can influence the density and occurrence of target and non-target animals, foraging resources, and thus bait uptake efficiency [56]. For this reason, Boyer et al. [56] recommended refining bait distribution based on habitat and target species; for example, forests fragmented by agricultural land are targeted for raccoons and field edges adjacent to forest patches for skunks. However, in a recent experimental study in which resource selection data were incorporated into bait distribution methods, bait uptake did not differ between treatment sites (where bait application was stratified according to estimated habitat use) and control sites (where baits were distributed uniformly along transects) [57]. Additional studies testing this concept are needed. The ORV delivery in the eastern US usually occurs in late summer to early fall, when young-of-year are moving, often in maternal family groups, and might encounter and consume baits. Juveniles are an important cohort to target, given that they represent a pulse of new susceptible individuals that are capable of driving transmission dynamics. During this time of year, natural food resources might become more limited, which increases the attractiveness of baits, and the vaccine baits are less likely to be impacted by extreme temperatures [58, 59]. This bait timing is also advantageous because maternal antibodies in juveniles would have waned by this time. Fry et al. [58] estimated the half-life of maternal antibodies to be 10.5 days post-weaning, and the results of this study also suggested (although not definitively) that the presence of maternal antibodies could interfere with a young raccoon’s ability to properly respond to oral vaccination. Similarly, juvenile foxes demonstrated an impaired immune response during a challenge with RABV, even after maternal antibodies were no longer detected [60]. Timing of raccoon baiting also considers the need to measure RABV neutralizing antibodies (rVNA) within the post-ORV recommended sampling window as the serologic metric for ORV performance sampling before winter weather commences in the north [61, 62]

Optimized flight-line spacing to ensure that target animals are likely to encounter baits is a critical strategy consideration. Owen et al. [63] estimated raccoon home range size in an undeveloped area as approximately 2.5 km^2^ (females) and 4 km^2^ (males) in a central Appalachian forest, but urban or suburban raccoon home ranges tend to be much smaller (e.g., average 0.2 km^2^) if animals do not have to travel far for resources [64]. In the past, ORV flight-line spacing in the US and Canada varied from 500 to 1500 meters (see Table 1). The baiting schemes are carefully chosen for each region in the ORV zone to ensure that sufficient land-area is covered to vaccinate as many target animals as possible, thus maximizing population-level immunity. The comparison between urban/suburban and rural bait delivery is also potentially confounded by different methods of bait distribution; helicopters and hand-baiting are used extensively in urban/suburban areas, whereas rural areas receive bait delivery by fixed-wing aircraft.

### Trap-vaccinate-release (TVR)

TVR is a resource-intensive management tool that uses parenteral vaccination to boost population immunity in emergency situations or where ORV baiting might not be feasible, such as in densely human-populated urban areas [65, 24, 66] or for skunks [24]. ONRAB protected captive skunks from rabies in an efficacy study [67] and also in a high density field application that eliminated the arctic fox variant from free-ranging skunks in Ontario [43]. However, results from other studies are contradictory because free-ranging skunks still demonstrate low population immunity in response to more commonly used ORV bait application densities (Table 1) [24, 50, 51, 68], which complicates management efforts because skunks are susceptible to spillover of raccoon rabies [11]. TVR is generally used to target animals in small areas (e.g., parks and urban neighborhoods) [69, 70, 66], but it is too labor intensive and expensive for broad-scale application [71]. This method is also used in contingency actions by the NRMP in response to ORV-zone breaches or threats of rabies spreading beyond existing zones [72].

In Central Park, New York, US, TVR was used to control an outbreak of raccoon rabies where ORV baiting was deemed infeasible, and local population reduction was publicly opposed [66]. In this case, rapid-response TVR likely prevented the virus from becoming enzootic in the Central Park raccoon population [66]. TVR was also used in an integrated point infection control (PIC) strategy in which raccoon rabies was detected across the St. Lawrence River in Ontario, Canada [71]. Rabies cases persisted until 2005, but Ontario is a much larger landscape to manage than New York, and the infection pressure from the US border states was constant [69, 71]. A TVR strategy was also used effectively in and around Flagstaff, Arizona following an outbreak of a bat rabies variant in striped skunks (*M*. *mephitis*) in 2001 [74, 73]. This multi-year TVR effort was deemed crucial for controlling this outbreak while ORV delivery systems and vaccine are being refined and optimized for skunks [24, 73].

### Population reduction

Population reduction and fertility control have long been considered important tools for reducing transmission of some density-dependent pathogens, although lethal management techniques are controversial among many stakeholders [75, 76]. Also, growing evidence demonstrates that population reduction over broad landscapes may not be achievable and that population reduction might not be effective for pathogen elimination in complex ecological systems, where social structure and contact dynamics are also influential, and transmission is not simply driven by density [77].

Local population reduction strategies can be reactive (in response to a specific outbreak), or proactive (independent of specific cases) [78, 77]. In the United Kingdom and Ireland, population reduction of Eurasian badgers (*Meles meles*) to reduce bovine tuberculosis (bTB, causative agent is *Mycobacterium bovis*) risk led to a decrease of the overall incidence of bTB in cattle (*Bos taurus*) within local population reduction areas. However, incidence became higher in untreated areas [79]. A potential explanation was that local population reduction within the treatment areas caused social perturbation and changed movement patterns among badger populations, enhancing the spread of bTB [80]. Although the two pathogens can be transmitted through different routes (bTB transmission can be indirect and RABV through direct contact), this example highlights a potential for unintended consequences following local population reduction.

Similarly, the potential consequences of local population reduction to manage disease in other wildlife species, such as raccoons, are not well understood [81]. The feasibility of local population reduction as a management tool might depend on the spatiotemporal scale of treatment and drivers of recolonization for the host species of concern. For example, three years after raccoon removal from agricultural habitat patches in Indiana, US, only 40% of the patches had recovered to preremoval densities. This was possibly driven by slow female recolonization and, thus, low site fidelity in immigrating males, ultimately suggesting that complete local population reduction might temporarily reduce the risk of density-dependent pathogen transmission [81]. In Ontario, Canada, sites subjected to reactive local population reduction in response to a raccoon rabies outbreak were not recolonized for ten months [82]. That local population reduction event provided a period of reduced raccoon density, which might offer emergency relief of risk of rabies spread [81, 82]. Population reduction might stimulate raccoon movements into control sites from areas where raccoon rabies is enzootic, or where rabies status unknown, exacerbating the intended outcome of reduced risk of rabies spread [81].

In Latin America, reactive attempts to reduce the local density of vampire bats (*Desmodus rotundus*) with an anticoagulant paste have not been effective in preventing or controlling rabies circulation [40, 83]. In Peru, bat exposure to RABV increased following sporadic local population reduction activities, possibly because these local bat population reduction efforts unintentionally targeted the wrong age-class (adults versus juveniles and sub-adults), or because the effort was not sufficient to impact RABV circulation in vampire bat populations [40]. Also, Streicker et al. [40] did not find evidence for density-dependent transmission in vampire bat colonies, an assumption that is also challenged for other rabies reservoirs [84]. In host populations in which transmission is frequency-dependent rather than density-dependent, or possibly a more complicated combination thereof, local population reduction alone is unlikely to be an effective disease management strategy [84]. Furthermore, subsequent changes in population structure, including if treated populations become more permissive to immigrants or if local population reduction activities trigger dispersal to new areas, could actually enhance rabies transmission [83].

### Fertility control

Fertility control offers potential alternatives to local population reduction or might be integrated with other methods to enhance the rabies control strategy. One potential advantage of some contraception methods, such as surgical spaying, is that population structure might not be disrupted. Fertility control can be achieved either surgically, chemically, or through immunocontraceptives. These different methods can vary in the temporal duration of efficacy. Contraception can be helpful as an alternative strategy to population reduction when a significant local population reduction would result in an influx of naïve hosts or when an increasing proportion of juvenile animals might increase transmission and epidemic risk [40, 83]. By applying contraception instead of population reduction, animals are not taken from the population, thus limiting immigration from other areas. Contraception can control the growth rate in a population, keeping the density below an epizootic threshold. Given the potential nontarget species impacts, other ecological concerns, and costs, orally delivered immunocontraceptives are not likely to be used on the landscape-scale to manage wildlife rabies; however, the tool might be useful when parenterally applied during TVR efforts. Surgical sterilization of dogs, coupled with vaccination, has been used to mitigate canine rabies in developing countries [84], with limited effectiveness. Also, at least two studies have evaluated concurrent administration of GonaCon and parenteral rabies vaccination; both vaccines induced rabies and gonadotropin-releasing hormone–specific immune responses in dogs in experimental settings [85, 86]. A conventional compartmental model designed for urban dogs predicted that a combination of vaccination and fertility control would achieve rabies elimination more quickly than vaccination alone, with less overall vaccine coverage required, although the model assumed homogeneous mixing of hosts [87].

## Modeling approaches to understand wildlife rabies

Mathematical models have long been used to predict and understand the dynamics of animal rabies [88, 89], and in this section, we review six general frameworks that have been used: simple epidemic models, host heterogeneity models, multi-host/multi-pathogen models, seasonal models, and spatial models [89, 90]. The general classifications used here are not mutually exclusive, and one model might fit into multiple categories. Examples of these methods are described below and in Table 2. Any of the frameworks mentioned here can be run as deterministic or stochastic models. Keeling and Rohani [90] describe deterministic models as “clockwork systems,” in which the conditions and parameter values in the model are fixed, thus leading to one (and the same) outcome each time the model is performed. Deterministic models can be useful to understand general trends within a system; however, there is no accounting for the random variation that is observed in real-world host/pathogen systems [90]. Alternatively, stochastic models incorporate chance and uncertainty by randomly choosing parameter values from probabilistic distributions, as defined by the model user [90]. With each repeated model iteration, the resulting values can change, and the aggregate output will reflect how parameter uncertainty affects the modeled system [90]. Also important to consider with all models, but not discussed in this paper, is the model validation procedure, which is used to determine which parameters contribute the most to uncertainty and variation in the total output. The identified parameters can then be further examined and might indicate empirical research needs [91].

**Table 2:** Summary of modeling approaches used to understand rabies dynamics in wildlife, conceptually structured as in Keeling and Rohani [90].

Method	Key Features	Pros	Cons	Examples
***Simple epidemic (deterministic)***	• Basic compartmental models (e.g., SIR, SIS, SEIR) • Assumes uniform mixing of the population • Population level only	• Computationally simple • Predictions of the model are easily interpreted • Control measures either succeed or not (no gray area)	• May not be biologically accurate	• Early red fox compartmental models [88]
***Simple epidemic (stochastic)***	• Explicit modeling of random events as part of model process/behavior • Population level only	• Estimates a more accurate presentation of stochastic nature of small populations and extinction processes • May be more appropriate for control programs which aim to reduce pathogens to extinction	• Computationally intensive	• Early models of the mid-Atlantic epizootic in raccoons [95, 96]
***Host heterogeneities (deterministic or stochastic)***	• Application of demographic cohorts which have quantified risk of transmission or susceptibility • Incorporate heterogeneous population mixing • Incorporate age-structure • Can use for individual and/or population level	• May help to identify individuals, social groups, or specific behaviors that influence rabies dynamics • May help to design more efficient, targeted measures for disease control	• Requires increased computational time and data needs for accurate parameterization	• Social network models [29, 38, 119] • Age-structured models [112] • Individual-based models [108]
***Multi-host/multi-pathogen (deterministic or stochastic)***	• Multiple host species affect the transmission cycle • Multiple pathogens impacting a host population (e.g. RABV and canine distemper virus) • Individual or population level	• Better understanding of multi-species (biodiversity) effects on transmission dynamics • Better understanding of how multiple circulating pathogens affect host demographic and transmission dynamics • Design more efficient targeted measures for disease control	• Increased computational time and data needs for parameterization • Need a thorough understanding of reservoir ecology and epizootiology	• Raccoon/skunk spillover model [26] • Dog/wild carnivore interactions and rabies dynamics in Africa [112, 113] • Rabies and canine distemper virus in African wild dogs [112]
***Seasonal (deterministic or stochastic)***	• Transmission and/or susceptibility of host has pronounced seasonality, within and/or across years • Individual or population level	• Better understanding of how life history impacts transmission dynamics (e.g. mating, migration, parturition)	• Increased computational time and data needs for parameterization	• Early red fox rabies models with seasonality [89] • Social Network Analysis: shows greater force of infection during spring (overwintered infections) and fall (dispersing juveniles) [38, 119] • Seasonal and multi-year rabies dynamics [158] • Impact of synchronized birth pulses [118, 120] • Individual-based models [108]
***Spatial (deterministic or stochastic)***	• Metapopulation models • Use of locality units (e.g., township, county, etc.) to circumscribe host population units (often because they match surveillance data) • Evaluation of natural (e.g., rivers, mountains) and man-made (e.g., roads, vaccination) barriers to spread • Individual or population level	• Focus on real landscapes of disease spread • Control points on the landscape may be easier to identify	• Increased computational time and data needs for parameterization • Requires relatively robust observation process	• Extension of red fox models to include spatial dynamics [92, 93] • Township, county level models [121, 122, 123] • Spatially explicit Individual-based model [108]

SIR, susceptible-infected-recovered; SIS, susceptible-infected-susceptible; SEIR, susceptible-exposed-infected-recovered

### Simple epidemic

Early simple deterministic epidemiological models were helpful for guiding the control of fox rabies in Europe. For example, Anderson et al. [88] incorporated red fox population biology into a simple deterministic, compartmental model to summarize the dynamics between host and pathogen interactions and to predict the effect of control methods such as culling and vaccination. Källen et al. [92] used a similar approach to model the spatial spread of rabies at the front of an epizootic wave. Murray [93] built on both of these models, combining spatial data and density estimates to more completely capture the epizootiology of fox rabies; this was instrumental in understanding the characteristics of epidemic wave fronts in fox rabies outbreaks in Europe and where control measures could best be applied. These simple models provided a foundation for the management of rabies in wildlife, ultimately leading to the development of more complex models that can accommodate stochasticity and behavioral factors. The newer models can be used to refine and adapt existing control programs, thus ensuring that resources are maximized while planning and managing rabies in changing and variable environments [94].

Childs et al. [95] tested a priori predictions of local rabies epizootic dynamics from a mathematical model [96] with empirical temporal data from the outbreak and found that model predictions matched the observed data, thus highlighting the value of predictive models. The use of simple epidemic models in the analysis of RABV is a good way to characterize general epidemiological features of an outbreak, such as prevalence, speed of spread, and incidence [92, 93, 94]. Also, these models can help users to understand the ecology of the RABV, or generate further hypotheses.

### Models to study host heterogeneities

Individual- and population-level heterogeneity in rabies hosts is important to understand for more accurate representation of host inter- and intrapopulation contact. One of the most difficult parameters to estimate in pathogen dynamic models is transmission rate, because it depends on knowledge of contact structure among individuals in a population and the relationship between individuals and the virus [97, 39, 38]. Although mapping and other spatial tools have been helpful to understand rabies spread over landscapes, they have limited value in explaining intra-population dynamics and interactions among individuals [29, 98]. Also, conventional compartmental models (e.g., susceptible-infectious-recovered), often assume homogeneous contact structure, but this might not always apply to wildlife populations that have variable degrees of sociality, disease-induced behavioral changes, or other drivers of spatial mixing [99, 29, 39].

Social network analysis (SNA) and network modelling are methods that have emerged recently to address questions of host behavior and pathogen transmission in human and animal health [39]. A common obstacle in raccoon rabies population dynamic modeling has been a firm understanding of the patterns of pathogen transmission between individual raccoons and social groups [29, 37], although limited data exist [97, 99, 100, 101, 102]. Gehrt and Fritzell [102] described significant home range overlap and shared denning behavior among related females in south Texas, US; however, genetic relatedness was not an important factor for the cohesion of male social groups [103]. A SNA study using proximity-collared raccoons in Illinois, US did not find that genetic relatedness had an effect on raccoon social network structure [37]. Perhaps this difference is reflective of variable raccoon density, urbanization, or geographic location, but it highlights the heterogeneity of host social ecology in different landscapes, especially where anthropogenic food sources or seasonal resource pulses are available [104]. Also, more contacts among unrelated individuals could indicate an increased rate of immigration into some populations and possibly a higher likelihood for the introduction of RABV [81].

Network studies suggest that raccoon populations are much more connected than initially recognized in suburban environments, where raccoons often occur at high densities [29]. Suburban raccoon populations have a high likelihood of rabies becoming enzootic following its introduction into the area [29, 38]. Also, dynamic network analysis of a suburban raccoon population predicted that the magnitude and speed of rabies spread are seasonally dependent [38]. The same study also predicted that ORV did not provide an effective barrier against rabies invasion until a vaccine-induced seroprevalence of ~85% was reached within the raccoon population, possibly reflecting the difficulty of ORV control and prospective elimination in metropolitan areas [24, 38]. Although network methods show promise in understanding rabies dynamics in highly structured populations, some challenges still exist. It is unknown what proportion of contacts based on proximity detection actually end in bites that would lead to transmission events [29, 39]. Additional contact network studies from raccoon populations and habitat types along the rural–urban gradient would be helpful to further evaluate the effectiveness of current ORV strategies, to improve our understanding of how raccoon movement and density affect rabies transmission along that gradient.

Contact network models are best suited to studying the patterns and effects of host behavior on RABV transmission and spread. Also, these models can also help to identify the characteristics of individuals who are potential superspreaders or otherwise play an important role in RABV ecology [105]. This information is useful because managers can implement control strategies based on the timing (e.g., seasonal, annual, etc.) or locations of the individuals identified by the model. Like most models, contact network models have some limitations when empirical data are applied, and as Craft and Caillaud [105] suggest, these models require a lot of data and it can take months or years to collect enough data to make robust inferences about a population [105]. Also, narrow windows of opportunity for data collection can limit inference to a certain timeframe, such as time of day or season [105]. For example, the availability of desired data might be limited by the frequency with which satellite collars take readings, or when radiotelemetry activities are performed by study personnel.

From a rabies management perspective, Individual-based models (IBMs) offer promise to simulate the population-level consequences of various control methods, including vaccination and local population reduction, while also accounting for landscape heterogeneity and contact structure within a raccoon population. IBMs have long been used for simulating bottom-up interactions in populations and communities and were first applied to forest-succession modeling [106, 107]. IBMs are most useful to identify individual heterogeneity that can affect an entire ecological system. In the 1990s, these models became popular to better understand fish and wildlife populations but eventually were extended to address problems in epidemiology and the understanding of disease agents, including raccoon RABV [106, 108]. Also, by running multiple iterations of a stochastic model, some of the uncertainty within the model outputs can be captured and sensitive parameters can be identified and studied in more detail. Currently, IBMs are used to make recommendations and inform decision-making in several areas of human enterprise, including the social sciences, advertising, natural resource management, and public health [109, 110, 111].

IBMs are intuitive for modeling rabies dynamics in wildlife because they can be structured to follow individual animals in a simulated population throughout their lifespans and behave according to biologically relevant parameters defined by the analyst. Biological and ecological events, such as mating, birth pulses, and pathogen invasions, can all be simulated, and the starting parameter values can be hypothetical (e.g., when no data are available) or taken from empirical reports in the literature [108, 109]. As discussed previously, individual movement (e.g., dispersal and translocation) and other behaviors (e.g., heterogeneous contact structure) can be important determinants of rabies persistence and spread, and are useful inputs for evaluating the effectiveness of various control strategies (e.g., the width of ORV zones) [108, 29].

### Multi-host/multi-pathogen

Multi-species and multi-pathogen models require a thorough understanding of the reservoir ecology and epizootiology, as well as intensive computational time, especially with a stochastic framework. These factors have limited their use in the past, however, as researchers continue to recognize host pathogens within a community ecology context, there is a growing need to develop more complex models to account for a greater number of ecological processes and features.

When multiple species of competent rabies hosts are present in an ecosystem, the ecology of these species, and the role of each in rabies dynamics, is crucial for unbiased modeling. Often, one species is considered to be a reservoir, while the occurrence of rabies infections in another can be the effect of pathogen spillover [112]. In this situation, it can be helpful to understand the interaction parameters, especially contact, among the multiple rabies hosts [113]. In the eastern US, the spillover of RABV from raccoons to skunks is an important factor to consider when interpreting models and planning management strategies [26]. Accounting for the dynamics of rabies in all potential host species may improve model accuracy and help managers to design more efficient and targeted disease control measures [26].

Individual animals might be concurrently infected with multiple pathogens, or multiple pathogens might be affecting population demographics at any given time [114], and it may be helpful to understand how rabies epizootics change when individuals and/or populations are being influenced by concurrent infectious disease processes. For example, canine distemper virus (CDV) is known to cause large changes in the population demographics of carnivores [112, 113, 115], and a rabies epizootic in a population already affected by CDV might have a different, or less predictable pattern of spread. Concurrent infections might also be detrimental to the affected population through additive mortality.

### Seasonal

Seasonality has long been recognized as an important factor in the dynamics of rabies transmission [116, 89]. Generally, seasonality can be studied at the individual or population level [89, 117]. From seasonal models, researchers can gain a better understanding of how life history events, such as mating, parturition, and juvenile dispersal, can affect transmission dynamics [118]. Several different model types have been used to detect and study seasonality, including social network models, simple epidemic models, and IBMs [38, 108, 118, 119, 120]. Clayton et al. [120] used a simple compartmental model to examine the role of birth pulses and temporal vaccine distribution on raccoon rabies epizootics. This study demonstrated that the coordination of birth pulses and vaccination timing are critical to rabies control and that general ordinary differential equation models can help a researcher to determine what control policies will limit the spread of rabies in raccoon populations [120]. Social network models have also been used to identify and study seasonality in raccoon rabies outbreaks [38, 119] and determined that social contact duration was a more important seasonal driver of raccoon rabies epidemics than birth pulses or social network shifts. These studies demonstrate that seasonality is another crucial consideration to account for while developing models of raccoon rabies dynamics.

### Spatial models

The raccoon rabies epizootic in the mid-Atlantic region of the US that began in the 1970s led to an exceptional outbreak of rabies in raccoon populations and prompted the study of spatial epizootic dynamics at the county level of mid-Atlantic and northeastern states [121].

The data from the mid-Atlantic raccoon rabies epizootic were used to parameterize predictive spatial models for raccoon rabies spread, which were then used to guide surveillance and strategic planning efforts at state and local levels [121, 122, 123, 124]. One alternative approach used phylodynamics based on the virus genetics to estimate the spatial spread of raccoon rabies [125], which was refined to explicitly incorporate landscape features in a more recent analysis [126].

The ongoing development of sophisticated spatial tools enables a progressive focus on rabies enzootic and epizootic dynamics over heterogeneous landscapes. Several mathematical and statistical methods are available to study raccoon and skunk habitat use, with implications for rabies occurrence and other health risks [108, 127, 128, 129, 130, 131, 132, 133, 134]. These methods can also help to elucidate how certain landscape features, such as roads, waterways, and habitat fragmentation, combine with raccoon habitat use to affect rabies-control strategies [56, 57, 108, 134, 135, 136], such as targeted baiting, TVR, or other control methods.

For example, IBMs have demonstrated that spatial heterogeneity in the landscape interacts with the effectiveness of vaccination [108]. Spatially explicit approaches with a grid-based IBM can allow for incorporation of landscape heterogeneity at both small and large scales, depending on the complexity of within-cell contact structure [109, 134, 136]. Simulated rabies-control measures such as vaccination, population reduction, and contraception can also be modeled. Habitat heterogeneity is important to consider for the planning of elimination strategies because managers can apply heavier control efforts in areas where vaccine coverage is inconsistent or implement additional research projects to study the phenomenon [108]. Rees et al. [108] demonstrated that spatial heterogeneity of a simulated landscape affects vaccination effectiveness, especially in good-quality homogeneous and low-quality heterogeneous habitats. Insufficient vaccination coverage is also predicted to be counterproductive by prolonging epizootics, increasing the total number of cases during an epizootic, and increasing the probability of rabies breaching a vaccination barrier [108]. Also from this study, moderate levels of seroconversion (>60%) were necessary to prevent epizootics and breaches of vaccine barrier zones [108].

Genetic data can also be applied to IBM simulations to understand the role of physical geography on mating and movement patterns; the infectious disease dynamics can also be inferred from the resulting estimations [134, 135, 136]. Quantification of the genetic population structure with IBMs directly demonstrated that landscapes provide structure for host population densities over space [134]. If it is known how the population genetics are structured over a landscape and where the natural barrier effects exist, these barriers can be augmented with ORV campaigns. Also, IBMs can provide a framework to quantify the strength of landscape barriers [134]. Integrating both landscape epidemiology and host/pathogen genetic data can help to identify routes of viral transmission and key regions in which to enact control measures [35, 125, 134, 135, 136].

Historically, a significant limitation of IBMs was the need for great computational intensity and more powerful computers to complete simulations in a time-efficient manner. Now that such computers exist, the primary problem that remains is getting high-quality data that are able to populate the many parameters used within an IBM framework.

The application of resource selection functions (RSFs), especially to guide stratified bait distribution, is an interesting concept and warrants further investigation [56, 57]. However, despite the widespread use of RSFs, model inference is sensitive to the size and spatial extent of the availability parameter. Interpretational bias is likely to be introduced if a sufficient availability sample is not selected, which is dependent on the ecology of the study species [137].

Maximum entropy (Maxent) models use machine learning methods to predict the geographic distributions of species and can also be applied to predict pathogen spread [138, 139]. This approach to ecological niche modeling can be advantageous because it requires presence-only species occurrence data to generate estimates of species distribution. Two major assumptions of this method, however, are that sampling is either random or representative throughout the area of interest and that detection probability is constant across sites [140]. If these assumptions are violated, possibly through opportunistic or haphazard sample collection, occurrence probability estimates might be misleading, resulting in poor inferences about species distributions [140].

Circuit theory models define areas of connectivity within landscapes [141], thus identifying potential areas where animal movements and contacts might be intensified, which could facilitate pathogen transmission. Circuit theory has been applied to rabies dynamics on both theoretical and real landscapes [142]. Using Maxent and circuit theory methods in tandem enables the identification of corridors in species-specific habitats; these are key areas of interest in which rabies-control methods can be applied to attempt to prevent the spatial spread of the virus [142]. Circuit theory can also be applied to predict patterns of gene flow and genetic differentiation and has been used to model the landscape genetics of raccoon RABV and reservoirs [134, 136].

## Looking forward in raccoon rabies management

Rabies management programs in Europe, Canada, and parts of the US demonstrated that rabies elimination is a feasible goal [24]. Regardless of the strategy used, all successful options will stress economic viability for the protection of human and animal health, a reality that cannot be ignored, even in a theoretical discussion [143, 144]. Kemere et al. [145] estimated that the maintenance of a large scale ORV barrier to prevent the westward spread of raccoon rabies was economically beneficial in every cost and spread scenario considered. Shwiff et al. [143] reported that for every dollar spent on a raccoon rabies program in Quebec, Canada, costs of $0.96 to $1.55 were prevented. Economic research also supports the case for elimination; Elser et al. [146] estimated that the successful raccoon rabies elimination program on Long Island, New York will financially benefit the state by $27 million by 2019. In the same study, the authors estimate that for every dollar spent on the program, $1.71 will be saved by 2019 [146]. A main driver of cost savings for New York is associated with reduced PEP and diagnostic testing of rabies suspect animals [146].

The maintenance of the current ORV zones and prevention of westward expansion of the raccoon rabies variant shows that large-scale vaccination with Raboral V-RG baits can manage the disease spread [24]. Moving forward with raccoon rabies elimination will require collaboration and strategic planning among experts from a variety of disciplines with a focus on multiple control methods, particularly in highly urbanized landscapes, where bait delivery can be especially challenging and raccoon densities are high [24]. Bait stations containing ORV baits in highly urban areas, such as in New York and Cape Cod, might help to deliver baits to raccoons, while limiting bait–human contact risks [147, 148]. The potential future broad-scale application of ONRAB in the US is a promising addition to ORV programs and the rabies management toolbox. Individual-based or other models that allow for species co-occurrence and cross-species transmission in various land-use scenarios could be helpful to understand the roles of raccoons and other species, such as skunks, in facilitating RABV persistence and to apply the most suitable rabies-control methods across habitats. Although not discussed in detail in this paper, genetic methods are highly valuable to understanding both host and pathogen evolution and ecology and should also be considered when refining landscape management strategies. Modeling methods are important for risk assessment, and are important components to raccoon rabies elimination and contingency action planning. Robust surveillance of raccoon populations is also necessary to ensure that the models are being used and revised within adaptive frameworks. Clearly, surveillance is critical for assessing the impacts of management actions on the ground. If model outputs do not match what is observed through surveillance, investigation into the discrepancies is warranted.

The combination of empirical field data and predictions from theoretical models has provided a modest understanding of the dynamics and control of RABV circulation in terrestrial wildlife. Model outcomes can be used to generate new research questions and indicate previously unrecognized research needs [91, 149]. For example, the NRMP, as a science-based program that regularly performs data-driven rabies management through the ORV program and other efforts, is working to parameterize predictive models with long-term program data from which future management decisions might be drawn. In addition, the collaborative nature of rabies management and research programs in North America highlights a true multidisciplinary approach [2] and emphasizes the value of decision-making frameworks based on both model predictions and empirical data analyses [149].

**Key learning points**

- The westward range expansion of the raccoon variant of the rabies virus is currently prevented by an oral rabies vaccine zone, and plans are underway to begin moving this zone eastward to eliminate raccoon rabies over the next 30 years.
- Multiple management options exist for raccoon rabies virus, most importantly the strategy of oral rabies vaccination targeting free-ranging mesocarnivores. Refinement of strategies might include enhanced targeting of specific habitats, landscapes, and/or demographic cohorts of animals.
- More recent modeling applications facilitate estimation of probabilities and uncertainties associated with simulated outcomes to management strategies through incorporation of model stochasticity.

**Top five papers**

1. Slate D, Algeo TP, Nelson KM, Chipman RB, Donovan D, Blanton JD, et al. Oral rabies vaccination in North America: Opportunities, complexities, and challenges. PLoS Negl Trop Dis. 2009;3(12):e549.
2. Rosatte RC. Evolution of wildlife rabies control tactics. Adv Virus Res. 2011;79:397–419.
3. Real LA, Russell C, Waller L, Smith D, Childs J. Spatial dynamics and molecular ecology of North American rabies. J Hered. 2005;96:253–260.
4. Rees EE, Pond BA, Tinline RR, Belanger D. Modelling the effect of landscape heterogeneity on the efficacy of vaccination for wildlife infectious disease control. J Appl Ecol. 2013;50:881–891.
5. Reynolds JH, Hirsch BT, Gehrt SD, Craft ME. Raccoon contact networks predict seasonal susceptibility to rabies outbreaks and limitations of vaccination. J Anim Ecol. 2015;84:1720–1731.

## References

[1] Rupprecht CE, Hanlon CA, Hemachudha T. Rabies re-examined. Lancet. 2002; 2: 101–109. 10.1016/s1473-3099(02)00287-6 12144896

[2] VerCauteren KC, Ellis C, Chipman R, DeLiberto T, Shwiff S, Slate D. Rabies in North America: A model of the One Health approach. In: Proceedings of the 14th Wildlife Damage Management Conference, Frey SN, editor. 2012; pp. 56–63.

[3] World Health Organization. 2015 [accessed 19 November, 2015]. http://www.who.int/rabies/en/.

[4] Hampson K, Coudeville L, Lembo T, Sambo M, Kieffer A, Attlan M, et al Estimating the global burden of endemic canine rabies. PLoS Negl Trop Dis. 2015; 9: e0003709 10.1371/journal.pntd.0003709 25881058 PMC4400070

[5] Smith JS, Orciari LA, Yager PA. Molecular epidemiology of rabies in the United States. Semin Virol. 1995; 6: 387–400.

[6] Hanlon CA, Childs JE, Nettles VF, the National Working Group on Rabies Control and Prevention. Article III: Rabies in wildlife. J Am Vet Med Assoc. 1999; 215: 1612–1619. 14575027

[7] Belotto A, Leanes LF, Schneider MC, Tamayo H, Correa E. Overview of rabies in the Americas. Virus Research. 2005; 111: 5–12. 10.1016/j.virusres.2005.03.006 15896398

[8] Rupprecht CE, Slate D. Rabies prevention and control: Advances and challenges In: Dietzgen RG, Kuzmin IV, editors. Rhabdoviruses: Molecular taxonomy, evolution, genomics, ecology, host-vector interactions, cytopathology, and control. Norfolk: Caister Academic Press, 2012 pp. 215–252.

[9] Anderson A, Shwiff SA. The cost of canine rabies on four continents. Transbound Emerg Dis. 2013; 62: 446–452. 10.1111/tbed.12168 24112194

[10] Christian KA, Blanton JD, Auslander M, Rupprecht CE. Epidemiology of rabies post-exposure prophylaxis–United States of America, 2006–2008. Vaccine. 2009; 27: 7156–7161. 10.1016/j.vaccine.2009.09.028 19925946

[11] Wallace RM, Gilbert AG, Slate D, Chipman R, Singh A, Wedd C, et al Right place, wrong species: A 20-year review of rabies virus cross species transmission among terrestrial mammals in the United States. PLoS ONE. 2014; 9: e107539 10.1371/journal.pone.0107539 25295750 PMC4189788

[12] Miller RS, Farnsworth ML, Malmberg JL. Diseases at the livestock-wildlife interface: status, challenges, and opportunities in the United States. Prev Vet Med. 2013; 110: 119–132. 10.1016/j.prevetmed.2012.11.021 23254245 PMC7127607

[13] Daszak P, Cunningham AA, Hyatt AD. Emerging infectious diseases of wildlife: Threats to biodiversity and human health. Science. 2000; 287: 443–449. 10642539 10.1126/science.287.5452.443

[14] Sterner RT, Smith GC. Modelling wildlife rabies: Transmission, economics, and conservation. Biol Cons. 2006; 131: 163–179.

[15] Callicott JB. The philosophical value of wildlife In: Armstrong SA, Botzler RG, editors. The Animal Ethics Reader. London: Rutledge; 2003.

[16] Taylor LH, Costa P, Briggs DJ. Public health management of humans at risk In: Jackson A, editor. Rabies: scientific basis of the disease and its management, 3^rd^ Edition Oxford: Elsevier; 2013 pp. 543–573.

[17] Kan VL, Joyce P, Benator D, Agnes K, Gill J, Irmler M, et al Risk assessment for healthcare workers after a sentinel case of rabies and review of the literature. Clin Infect Dis. 2015; 60: 341–348. 10.1093/cid/ciu850 25352591

[18] Middleton D, Johnson KO, Rosatte RC, Hobbs JL, Moore SR, Rosella L, Crowcroft NS. Human rabies post-exposure prophylaxis and animal rabies in Ontario, Canada, 2001–2012. Zoonoses Public Health. 2015; 62: 356–364. 10.1111/zph.12155 25244148

[19] McLean RG. Raccoon rabies In: Baer GM, editor. The natural history of rabies, Vol. II New York: Academic Press; 1975 pp. 53–77.

[20] Rupprecht CE, Smith JS. Raccoon rabies: the re-emergence of an epizootic in a densely populated area. Semin. Virol. 1994; 5: 155–164.

[21] Nettles VF, Shaddock JH, Sikes RK, Reyes CR. Rabies in translocated raccoons. Amer J Publ Health. 1979; 69: 601–602. 10.2105/ajph.69.6.601 PMC1618975 443502

[22] Rupprecht CE, Glickman LT, Spencer PA, Wiktor TJ. Epidemiology of rabies virus variants–differentiation using monoclonal-antibodies and discriminant-analysis. Am J Epidemiol. 1987; 126: 298–309. 3300280 10.1093/aje/126.2.298

[23] McQuiston JH, Yager PA, Smith JS, Rupprecht CE. Epidemiologic characteristics of rabies virus variants in dogs and cats in the United States, 1999. J Am Vet Med Assoc. 2001; 218: 1939–1942. 11417737 10.2460/javma.2001.218.1939

[24] Slate D, Algeo TP, Nelson KM, Chipman RB, Donovan D, Blanton JD, et al Oral rabies vaccination in North America: Opportunities, complexities, and challenges. PLoS Negl Trop Dis. 2009; 3: e549 10.1371/journal.pntd.0000549 20027214 PMC2791170

[25] Müller TF, Freuling CM, Wysocki P, Roumiantzeff M, Freney J, Mettenleiter TC, Vos AC. Terrestrial rabies control in the European Union: Historical achievements and challenges ahead. Vet J. 2015; 203: 10–17. 10.1016/j.tvjl.2014.10.026 25466578

[26] Guerra MA, Curns AT, Rupprecht CE, Hanlon CA, Krebs JW, Childs JE. Skunk and raccoon rabies in the eastern United States: temporal and spatial analysis. Emerg Inf Dis. 2003; 9: 1143–1150. 10.3201/eid0909.020608 PMC3016792 14519253

[27] Chipman R, Slate D, Rupprecht C, Mendoza M. Downside risk of wildlife translocation. In: Dodet B, Fooks AR, Muller T, Tordo N, and the Scientific and Technical Department of the OIE (editors). Towards the elimination of rabies in Eurasia. Dev Biol 2008. 131: 223–232. 18634483

[28] Randa LA, Yunger JA. Carnivore occurrence along an urban-rural gradient: a landscape level analysis. J Mammal. 2006; 87: 1154–1164.

[29] Hirsch BT, Prange S, Hauver SA, Gehrt SD. Raccoon social networks and the potential for disease transmission. PLoS ONE. 2013a; 8: e75830. 24130746 10.1371/journal.pone.0075830 PMC3794951

[30] Totton SC, Rosatte RC, Tinline RR, Bigler LL. Seasonal home ranges of raccoons, *Procyon lotor*, using a common feeding site in rural eastern Ontario: Rabies management implications. Can Field Nat. 2004; 118: 65–71.

[31] Houle M, Fortin D, Mainguy J, Canac-Marquis P. Landscape composition and structure influence the abundance of mesopredators: implications for the control of the raccoon (*Procyon lotor*) variant of rabies. Can J Zool. 2011; 89: 1107–1116.

[32] Beasley JC, Olson ZH, Dharmarajan G, Eagan TS II, Rhodes OE Jr. Spatio-temporal variation in the demographic attributes of a generalist mesopredator. Landscape Ecol. 2011; 26: 937–950.

[33] Puskas RB, Fischer JW, Swope CB, Dunbar MR, McLean RG, Root JJ. Raccoon (*Procyon lotor*) movements and dispersal associated with ridges and valleys of Pennsylvania: Implications for rabies management. VectorBorne Zoonotic Dis. 2010; 10: 1043–1048. 10.1089/vbz.2009.0079 20455781

[34] Côté H, Garant D, Robert K, Mainguy J, Pelletier F. Genetic structure and rabies spread potential in raccoons: the role of landscape barriers and sex-biased dispersal. Evol Appl. 2012; 5: 393–404. 10.1111/j.1752-4571.2012.00238.x 25568059 PMC3353356

[35] Rioux Paquette SR, Talbot B, Garant D, Mainguy J, Pelletier F. Modelling the dispersal of the two main hosts of the raccoon rabies variant in heterogeneous environments with landscape genetics. Evol App. 2014; 7: 734–749. 10.1111/eva.12161 PMC4227855 25469156

[36] Smith DL, Waller LA, Russell CA, Childs JE, Real LA. Assessing the role of long-distance translocation and spatial heterogeneity in the raccoon rabies epidemic in Connecticut. Prev Vet Med. 2005; 71: 225–240. 10.1016/j.prevetmed.2005.07.009 16153724 PMC7114108

[37] Hirsch BT, Prange S, Hauver S, Gehrt SD. Genetic relatedness does not predict raccoon social network structure. Anim Behav. 2013b; 85: 463–470.

[38] Reynolds JH, Hirsch BT, Gehrt SD, Craft ME. Raccoon contact networks predict seasonal susceptibility to rabies outbreaks and limitations of vaccination. J Anim Ecol. 2015; 84: 1720–1731. 10.1111/1365-2656.12422 26172427

[39] Craft ME. Infectious disease transmission and contact networks in wildlife and livestock. Phil Trans R Soc B. 2015; 370; 20140107. 10.1098/rstb.2014.0107 PMC4410373 25870393

[40] Streicker DG, Recuenco S, Valderrama W, Benavides JG, Vargas I, Pachecho V, Ecological and anthropogenic drivers of rabies exposure in vampire bats: implications for transmission and control. Proc R Acad B. 2012; 279: 3384–3392. 10.1098/rspb.2012.0538 PMC3396893 22696521

[41] Morters MK, Restif O, Hampson K, Cleaveland S, Wood JLN, Conlan AJK. Evidence-based control of canine rabies: a critical review of population density reduction. J Anim Ecol. 2013; 82: 6–14. 10.1111/j.1365-2656.2012.02033.x 23004351 PMC3579231

[42] Freuling CM, Hampson K, Selhorst T, Schröder R, Meslin F.X., Mettenleiter T.C., et al The elimination of fox rabies from Europe: determinants of success and lessons for the future. Phil Trans R Soc B. 2013; 368: 20120142 10.1098/rstb.2012.0142 23798690 PMC3720040

[43] Rosatte RC, Donovan D, Davies JC, Brown L, Allan M, von Zuben V, et al High-Density baiting with ONRAB® rabies vaccine baits to control Arctic-variant rabies in striped skunks in Ontario, Canada. J Wildl Dis. 2011; 47: 495–465. 10.7589/0090-3558-47.2.459 21441200

[44] Sidwa TJ, Wilson PJ, Moore GM, Oertli EH, Hicks BN, Rohde RE, et al Evaluation of oral rabies vaccination programs for control of rabies epizootics in coyotes and gray foxes: 1995–2003. J Am Vet Med Assoc. 2005; 227: 785–792. 16178403 10.2460/javma.2005.227.785

[45] Velasco-Villa A, Reeder SA, Oricari LA, Yager PA Franka R, Blanton JD, Zuckero L, Hunt P, Oertli EH, Robinson LE, Rupprecht CE. Enzootic rabies elimination from dogs and reemergence in wild terrestrial carnivores, United States. Emerg Inf Dis. 2008; 14: 1849–1854. 10.3201/eid1412.080876 PMC2634643 19046506

[46] United States Department of Agriculture. 2008 [accessed 13 August, 2015]. North American Rabies Management Plan. http://www.aphis.usda.gov/wildlife_damage/oral_rabies/downloads/Final%20NARMP%209-30-2008%20(ENGLISH).pdf.

[47] Sterner RT, Meltzer MI, Shwiff SA, Slate D. Tactics and economics of wildlife oral rabies vaccination, Canada and the United States. Emerg Inf Dis. 2009; 15: 1176–1184. 10.3201/eid1508.081061 PMC2815952 19757549

[48] Müller TF, Schröder R, Wysocki P, Mettenleiter TC, Freuling CM. Spatio-temporal use of oral rabies vaccines in fox rabies elimination programmes in Europe. PLoS Negl Trop Dis.2015; 9: e0003953 10.1371/journal.pntd.0003953 26280895 PMC4539187

[49] Rosatte RC. Evolution of wildlife rabies control tactics. Adv Virus Res. 2011b; 79 397–419. 21601057 10.1016/B978-0-12-387040-7.00019-6

[50] Slate D, Chipman RB, Algeo TP, Mills SA, Nelson KM, Croson CK, Dubovi EJ, VerCauteren KC, Renshaw RW, Atwood T, Johnson S, Rupprecht CE. Safety and immunogenicity of Ontario Rabies Vaccine Bait (ONRAB) in the first US field trial in raccoons (*Procyon lotor*). J Wildl Dis. 2014; 50: 582–595. 10.7589/2013-08-207 24807178

[51] Fehlner-Gardiner C, Rudd R, Donovan D, Slate D, Kempf L, Badcock J. Comparing ONRAB® and RABORAL V-RG® oral rabies vaccine field performance in raccoons and striped skunks, New Brunswick, Canada and Maine, USA. J Wildl Dis. 2012; 48: 157–167. 10.7589/0090-3558-48.1.157 22247384

[52] Mainguy J, Fehlner-Gardiner C, Slate D, Rudd RJ. Oral rabies vaccination in raccoons: comparison of ONRAB® and RABORAL V-RG® vaccine-bait field performance in Québec, Canada and Vermont, USA. J Wildl Dis. 2013; 49: 190–193. 10.7589/2011-11-342 23307388

[53] Rupprecht CE, Smith JS, Fekadu M, Childs JE. The ascension of wildlife rabies: a cause for public health concern or intervention? Emerg Inf Dis. 1995; 1: 107–114. 10.3201/eid0104.950401 PMC2626887 8903179

[54] Ramey PC, Blackwell BF, Gates RJ, Slemons RD. Oral rabies vaccination of a northern Ohio raccoon population: Relevance of population density and prebait serology. J Wildl Dis. 2008; 44: 553–568. 10.7589/0090-3558-44.3.553 18689640

[55] Beasley JC, Beatty WS, Atwood TC, Johnson SR, Rhodes OE Jr. A comparison of methods for estimating raccoon abundance: Implications for disease vaccination programs. J Wildl Manage 2012; 76: 1290–1297.

[56] Boyer JP, Canac-Marquis P, Guérin D, Mainguy J, Pelletier F. Oral vaccination against raccoon rabies: landscape heterogeneity and timing of distribution influence wildlife contact rates with the ONRAB vaccine bait. J Wildl Dis. 2011; 47: 593–602. 10.7589/0090-3558-47.3.593 21719823

[57] Beasley JC, Atwood TC, Byrne ME, Vercauteren KC, Johnson SR, Rhodes OE Jr. A behaviorally-explicit approach for delivering vaccine baits to mesopredators to control epizootics in fragmented landscapes. PLoS ONE. 2015; 10: e113206. 10.1371/journal.pone.0113206 PMC4294636 25587900

[58] Fry TL, VanDalen KK, Shriner SA, Moore SM, Hanlon CA, VerCauteren KC. Humoral immune response to oral rabies vaccination in raccoon kits: Problems and implications. Vaccine. 2013; 31: 2811–2815. 10.1016/j.vaccine.2013.04.016 23602534

[59] Parsons AW, Simons TR, O’Connell AF, Stoskopf MK. Demographics, diet, movements, and survival of an isolated, unmanaged raccoon *Procyon lotor* (Procyonidae, Carnivora) population on the Outer Banks of North Carolina. Mammalia. 2013; 77: 21–30.

[60] Müller TF, Schuster P, Vos AC, Selhorst T, Wenzel UD, Neubert AM. Effect of maternal immunity on the immune response to oral vaccination against rabies in young foxes. Am J Vet Res. 2001; 62: 1154–1158. 11453495 10.2460/ajvr.2001.62.1154

[61] Hanlon CA, Niezgoda M, Hamir AN, Schumacher C, Koprowski H, Rupprecht CE. First North American field release of a vaccinia-rabies glycoprotein recombinant virus. J Wildl Dis. 1998; 34: 228–239. 10.7589/0090-3558-34.2.228 9577769

[62] Brown LJ, Rosatte RC, Fehlner-Gardiner C, Taylor JS, Davies JC, Donovan D. Immune response and protection in raccoons *(Procyon lotor)* following consumption of baits containing ONRAB®, a human adenovirus rabies glycoprotein recombinant vaccine. J Wildl Dis. 2012; 48: 1010–1020. 10.7589/2012-01-023 23060502

[63] Owen SF, Berl JL, Edwards JW, Ford WM, Wood PB. Raccoon spatial requirements and multi-scale habitat selection within an intensively managed central Appalachian forest. Amer Mid Nat. 2015; 174: 87–95.

[64] Berentsen AR, Dunbar MR, Fitzpatrick CE, Walter WD. Spatial ecology of urban raccoons in northeastern Ohio: Implications for oral rabies vaccination. Prairie Naturalist. 2013; 45: 39–45.

[65] Rosatte RC, Power MJ, MacInnes CD, Campbell JB. Trap-vaccinate-release and oral vaccination for rabies control in urban skunks, raccoons, and foxes. J Wildl Dis. 1992; 28: 562–571. 10.7589/0090-3558-28.4.562 1474654

[66] Slavinski S, Humberg L, Lowney M, Simon R, Calvanese N, Bregman B, et al Trap-vaccinate-release program to control raccoon rabies, New York, USA. Emerg Inf Dis. 2012; 18: 1170–1172. 10.3201/eid1807.111485 PMC3376792 22709617

[67] Brown LJ, Rosatte RC, Fehlner-Gardiner C, Ellison JA, Jackson FR, Bachmann P, et al Oral vaccination and protection of striped skunks (*Mephitis mephitis*) against rabies using ONRAB®. Vaccine. 2014; 32: 3675–3679. 10.1016/j.vaccine.2014.04.029 24814554

[68] Mainguy J, Rees EE, Canac-Marquis P, Bélanger D, Fehlner-Gardiner C, Séguin G, Larrat S, Landry F, Côté N. Oral rabies vaccination of raccoons and striped skunks with ONRAB® baits: multiple factors influence field immunogenicity. J Wildl Dis. 2012; 48: 979–990. 10.7589/2011-12-316 23060499

[69] Rosatte R, MacDonald E, Sobey K, Donovan D, Bruce L, Allan M, et al The elimination of raccoon rabies from Wolfe Island, Ontario: Animal density and movements. J Wildl Dis. 2007b; 43: 242–250. 17495308 10.7589/0090-3558-43.2.242

[70] Rosatte RC, Donovan D, Allan M, Bruce L, Buchanan T, Sobey K, et al The control of raccoon rabies in Ontario, Canada: proactive and reactive tactics, 1994–2007. J Wildl Dis. 2009; 45: 772–784. 10.7589/0090-3558-45.3.772 19617488

[71] Rosatte R, Donovan D, Allan M, Howes LA, Silver A, Bennett K, et al Emergency response to raccoon rabies introduction into Ontario. J Wildl Dis. 2001; 37: 265–279. 10.7589/0090-3558-37.2.265 11310877

[72] Rees EER, Bélanger D, Lelièvre F, Coté N, Lambert L. Targeted surveillance of raccoon rabies in Québec, Canada. J Wildl Manage. 2011; 75: 1406–1416.

[73] United States Department of Agriculture. National Rabies Management Program Summary Report 2010. 2010 [accessed 5 November, 2015]. https://www.aphis.usda.gov/wildlife_damage/oral_rabies/downloads/NationalReport_2010.pdf.

[74] Leslie MJ, Messenger S, Rohde RE, Smith J, Cheshier R, Hanlon C, Rupprecht CE. Bat-associated rabies virus in skunks. Emerg Infect Dis. 2006; 12: 1274–1277. 10.3201/eid1208.051526 16965714 PMC3291214

[75] Rosatte RC, Pybus MJ, Gunson JR. Population reduction as a factor in the control of skunk rabies in Alberta. J Wildl Dis. 1986; 22: 459–467. 3503130 10.7589/0090-3558-22.4.459

[76] Dandy N, Ballantyne S, Moseley D, Gill R, Quine C, Van Der Wal R. Exploring beliefs behind support for and opposition to wildlife management methods: a qualitative study. Eur J Wildl Res. 2012; 58: 695–706.

[77] Bolzoni L, Tessoni V, Groppi M, De Leo GA. React or wait: which optimal culling strategy to control infectious diseases in wildlife. J Math Biol. 2014; 69: 1001–1025. 10.1007/s00285-013-0726-y 24057080

[78] Donnelly CA, Woodroffe R, Cox DR, Bourne FJ, Cheeseman CL, Clifton-Hadley RS. Positive and negative effects of widespread badger culling on tuberculosis in cattle. Nature. 2006; 439: 843–846. 10.1038/nature04454 16357869

[79] Donnelly CA, Wei G, Johnston WT, Cox DR, Woodroffe, Bourne FJ, et al Impacts of widespread badger culling on cattle tuberculosis: concluding analyses from a large-scale field trial. Int J Infect Dis. 2007; 11: 300–308. 10.1016/j.ijid.2007.04.001 17566777

[80] Carter SP, Delahay RJ, Smith GC, Macdonald DW, Riordan P, Etherington TR, et al Culling-induced social perturbation in Eurasian badgers *Meles meles* and the management of TB in cattle; an analysis of a critical problem in applied ecology. Proc R Soc B. 2007; 274: 2769–2777. 10.1098/rspb.2007.0998 17725974 PMC2279223

[81] Beasley JC, Olson ZH, Beatty WS, Dharmarajan G, Rhodes OE Jr. Effects of culling on mesopredator population dynamics. PLoS ONE. 2013; 8: e58982 10.1371/journal.pone.0058982 23527065 PMC3604110

[82] Rosatte R, Sobey K, Donovan D, Allan M, Bruce L, Buchanan T, et al Raccoon density and movements after population reduction to control rabies. J Wildl Manage. 2007a; 71: 2372–2378.

[83] Blackwood JC, Streicker DE, Altizer S, Rohani P. Resolving the roles of immunity, pathogenesis, and immigration for rabies persistence in vampire bats. Proc Nat Acad Sci. 2013; 110: 20837–20842. 10.1073/pnas.1308817110 24297874 PMC3870737

[84] Reece JF, Chawla SK. Control of rabies in Jaipur, India, by the sterilization and vaccination of neighbourhood dogs. Vet Rec. 2006; 159: 379–383. 16980523 10.1136/vr.159.12.379

[85] Bender SC, Bergman DL, Wenning KM, Miller LA, Slate D, Jackson FR, et al No adverse effects of simultaneous vaccination with the immunocontraceptive GonaCon™ and a commercial rabies vaccine on rabies virus neutralizing antibody production in dogs. Vaccine. 2009; 27: 7210–7213. 10.1016/j.vaccine.2009.09.026 19925955

[86] Vargas-Pino F, Gutierrez-Cedillo V, Canales-Vargas EJ, Gress-Ortega LR, Miller LA, Rupprecht CE, et al Concomitant administration of GonaCon™ and rabies vaccine in female dogs (*Canis familaris*) in Mexico. Vaccine. 2013; 31: 4442–4447. 10.1016/j.vaccine.2013.06.061 23871822

[87] Carroll MJ, Singer A, Smith GC, Cowan DP, Massei G. The use of immunocontraception to improve rabies eradication in urban dog populations. Wildl Res. 2010; 37: 676–687.

[88] Anderson RM, Jackson HC, May RM, Smith AM. Population dynamics of fox rabies in Europe. Nature. 1981; 289: 765–771. 7464941 10.1038/289765a0

[89] Bacon PJ. Population dynamics of rabies in wildlife. London: Academic Press, 1985.

[90] Keeling MJ, Rohani P. Modeling infectious diseases in humans and animals. Princeton: Princeton University Press, 2007.

[91] Restif O, Hayman DTS, Pulliam JRC, George DB, Luis AD, Cunningham AA. Model-guided fieldwork: practical guidelines for multidisciplinary research on wildlife ecological and epidemiological dynamics. Ecol Lett. 2012; 15: 1083–1094. 10.1111/j.1461-0248.2012.01836.x 22809422 PMC3466409

[92] Källen A, Arcuri P, Murray JD. A simple model for the spatial spread and control of rabies. J Theor Biol. 1985; 116: 377–393. 4058027 10.1016/s0022-5193(85)80276-9

[93] Murray JD. Modeling the spread of rabies. Amer Sci. 1987; 75: 280–284.

[94] Barlow ND. The ecology of wildlife disease control: simple models revisited. J Appl Ecol. 1996; 33: 303–114.

[95] Childs JE, Curns AT, Dey ME, Real LA, Feinstein L, Bjørnstad ON, et al Predicting the local dynamics of epizootic rabies among raccoons in the United States. Proc Nat Acad Sci. 2000; 97: 13666–13671. 10.1073/pnas.240326697 11069300 PMC17633

[96] Coyne MJ, Smith G, McAllister FE. Mathematic model for the population biology of rabies in raccoons in the Mid-Atlantic States. Am J Vet Res. 1989; 50: 2148–2154. 2610445

[97] Totton SC, Tinline RR, Rosatte RC, Bigler LL. Contact rates of raccoons (*Procyon lotor*) at a communal feeding site in rural eastern Ontario. J Wildl Dis. 2002; 38: 313–319. 10.7589/0090-3558-38.2.313 12038131

[98] Barton HD, Gregory AJ, Davis R, Hanlon CA, Wisely SM. Contrasting landscape epidemiology of two sympatric rabies virus strains. Mol Ecol. 2010; 19: 2725–2738. 10.1111/j.1365-294X.2010.04668.x 20546130

[99] Rosatte R, Sobey K, Donovan D, Bruce L, Allan M, Silver A, et al Behavior, movements, and demographics of rabid raccoons in Ontario, Canada: Management implications. J Wildl Dis. 2006; 42: 589–605. 10.7589/0090-3558-42.3.589 17092890

[100] Prange S, Gehrt SD, Hauver S. Frequency and duration of contacts between free-ranging raccoons: uncovering a hidden social system. J Mammal. 2011; 92: 1331–1342.

[101] Robert K, Garant D, Pelletier F. Keep in touch: Does spatial overlap correlate with contact frequency? J Wildl Manage. 2012; 76: 1670–1675.

[102] Gehrt SD, Fritzell EK. Duration of familial bonds and dispersal patterns for raccoons in south Texas. J Mammal. 1998; 79: 859–872.

[103] Gehrt SD, Gergits WF, Fritzell EK. Behavioral and genetic aspects of male social groups in raccoons. J Mammal. 2008; 89: 1473–1480.

[104] Becker DJ, Streicker DG, Altizer S. Linking anthropogenic resources to wildlife-pathogen dynamics: a review and meta-analysis. Ecol Lett. 2015; 18: 483–495. 10.1111/ele.12428 25808224 PMC4403965

[105] Craft ME, Caillaud D. Network models: An underutilized tool in wildlife epidemiology? Interdiscip Perspect Infect Dis. 2011; 2011: 676949 10.1155/2011/676949 21527981 PMC3063006

[106] Jeltsch F, Muller MS, Grimm V, Wissel C, Brandl R. Pattern formation triggered by rare events: lessons from the spread of rabies. Proc R Soc Lond B. 1997; 264: 495–503. 10.1098/rspb.1997.0071 PMC1688393 9149424

[107] Grimm V, Revilla E, Berger U, Jeltsch F, Mooij WM, Railsback SF, et al Pattern-oriented modeling of agent-based complex systems: lessons from ecology. Science. 2005; 310: 987–991. 10.1126/science.1116681 16284171

[108] Rees E.E., Pond B.A., Tinline R.R., Belanger D. 2013 Modelling the effect of landscape heterogeneity on the efficacy of vaccination for wildlife infectious disease control. J Appl Ecol. 50: 881–891.

[109] DeAngelis DL, Grimm V. Individual-based models in ecology after four decades. F1000Prime Rep. 2014; 6: 39 10.12703/P6-39 24991416 PMC4047944

[110] Abuelezam NN, Rough K, Seage GR III. Individual-based simulation models of HIV transmission: Reporting quality and recommendations. PLoS ONE. 2013; 8: e75624 10.1371/journal.pone.0075624 24098707 PMC3787035

[111] Wood KA, Stillman RA, Goss-Custard JD. Co-creation of individual-based models by practitioners and modellers to inform environmental decision-making. J Appl Ecol. 2015; 52: 810–815.

[112] Prager KC, Mazet JAK, Dubovi EJ, Frank LG, Munson L, Wagner AP, Woodroffe R. Rabies virus and canine distemper virus in wild and domestic carnivores in Northern Kenya: Are domestic dogs the reservoir? 2012 Ecohealth; 9: 483–498. 10.1007/s10393-013-0815-9 23459924

[113] Woodroffe R, Prager KC, Munson L, Conrad PA, Dubovi EJ, Mazet JA. Contact with domestic dogs increases pathogen exposure in endangered African wild dogs (*Lycaon pictus*). 2012 PLoS ONE; 7: e30099 10.1371/journal.pone.0030099 22238695 PMC3253127

[114] Hamir AN, Summers BA, Rupprecht CE. Concurrent rabies and canine distemper encephalitis in a raccoon (*Procyon lotor*). 1998 J Vet Diagn Invest; 10: 194–196. 10.1177/104063879801000218 9576354

[115] Nouvellet P, Donnelly CA, De Nardi M, Rhodes CH, De Benedictis P, et al Rabies and Canine Distemper Virus epidemics in the red fox population of Northern Italy (2006–2010). PLoS ONE. 2013; 8: e61588 10.1371/journal.pone.0061588 23630599 PMC3632604

[116] George DB, Webb CT, Farnsworth ML, O’Shea TJ, Bowen RA, Smith DL, et al Host and viral ecology determine bat rabies seasonality and maintenance. Proc Nat Acad Sci. 2011; 108: 10208–10213. 10.1073/pnas.1010875108 21646516 PMC3121824

[117] Altizer S, Dobson A, Hosseini P, Pascual M, Rohani P. Seasonality and the dynamics of infectious diseases. Ecol Lett. 2006; 9: 467–484. 10.1111/j.1461-0248.2005.00879.x 16623732

[118] Peel AJ, Pulliam JR, Luis AD, Plowright RK, O’Shea TJ, Hayman DT, et al The effect of seasonal birth pulses on pathogen persistence in wild mammal populations. Proc Biol Sci. 2014; 281: 1786. 10.1098/rspb.2013.2962 PMC4046395 24827436

[119] Hirsch BT, Reynolds SD, Gehrt SD, Craft ME. Which mechanisms drive seasonal rabies outbreaks in raccoons? A test using dynamic social network models. J Appl Ecol. 2016; 53: 804–813.

[120] Clayton T, Duke-Sylvester S, Gross LJ, Lenhart S, Real LA. Optimal control of a rabies epidemic model with a birth pulse. 2011 J Biol Dyn; 4: 43–58. 10.1080/17513750902935216 PMC3060792 21423822

[121] Smith DL, Lucey B, Waller LA, Childs JE, Real LA. Predicting the spatial dynamics of rabies epidemics on heterogeneous landscapes. Proc Nat Acad Sci. 2002; 99: 3668–3672. 10.1073/pnas.042400799 11904426 PMC122581

[122] Russell CA, Smith DL, Waller LA, Childs JE, Real LA. *A priori* prediction of disease invasion dynamics in a novel environment. Proc R Soc Lond. B. 2004; 271: 21–25. 10.1098/rspb.2003.2559 PMC1691560 15002767

[123] Russell CA, Smith DL, Childs JE, Real LA. Predictive spatial dynamics and strategic planning for raccoon rabies emergence in Ohio. PLoS Biol. 2005; 3: e88 10.1371/journal.pbio.0030088 15737065 PMC1054883

[124] Real L. A., Russell C., Waller L., Smith D., Childs J. Spatial dynamics and molecular ecology of North American rabies. J Hered. 2005; 96: 253–260. 10.1093/jhered/esi031 15677743

[125] Biek R, Henderson JC, Waller LA, Rupprecht CE, Real LA. A high-resolution genetic signature of demographic and spatial expansion in epizootic rabies virus. Proc Nat Acad Sci. 2007; 104: 7993–7998. 10.1073/pnas.0700741104 17470818 PMC1876560

[126] Dellicour S, Rose R, Pybus OG. Explaining the geographic spread of emerging epidemics: a framework for comparing viral phylogenies and environmental landscape data. BMC Bioinform. 2016; 17: 82. 10.1186/s12859-016-0924-x PMC4750353 26864798

[127] Chow TE, Gaines KF, Hodgson ME, Wilson MD. Habitat and exposure modelling for ecological risk assessment: A case study for the raccoon on the Savannah River site. Ecol Modell. 2005; 189: 151–167.

[128] Beasley JC, DeVault TL, Retamosa MI, Rhodes OE Jr. A hierarchical analysis of habitat selection by raccoons in northern Indiana. J Wildl Manage. 2007; 71: 1125–1133.

[129] Arjo WM, Fisher CE, Armstrong J, Boyd F, Slate D. Effects of natural barriers and habitat on the western spread of raccoon rabies in Alabama. J. Wildl. Manage. 2008; 72: 1725–1735.

[130] Recuenco S, Blanton JD, Rupprecht CE. A spatial model to forecast raccoon rabies emergence. Vector Borne Zoonotic Dis. 2012; 12: 126–136. 10.1089/vbz.2010.0053 21995266

[131] Ramey CA, Mills KW, Fischer JW, McLean RG, Fagerstone KA, Engeman RM. Graphically characterizing the movement of a rabid striped skunk epizootic across the landscape in a northwestern Wyoming. Ecohealth. 2013; 10: 246–256. 10.1007/s10393-013-0853-3 23812724

[132] Tardy O, Masse A, Pelletier F, Mainguy J, Fortin D. Density-dependent functional responses in habitat selection by two hosts of the raccoon rabies virus variant. Ecosphere. 2014; 5: 132.

[133] Raghavan RK, Hanlon CA, Goodin DG, Davis R, Moore M, Moore S, Anderson GA. Bayesian spatiotemporal pattern and eco-climatological drivers of striped skunk rabies in the North Central Plains. PLoS Negl Trop Dis. 2016; 10: e0004632 10.1371/journal.pntd.0004632 27127994 PMC4851358

[134] Rees EE, Pond BA, Cullingham CI, Tinline RR, Ball D, Kyle CJ, White BN. Landscape modelling spatial bottlenecks: implications for raccoon rabies disease spread. Biol Lett. 2009; 5: 387–390. 10.1098/rsbl.2009.0094 19324623 PMC2679935

[135] Cullingham CI, Kyle CJ, Pond BA, Rees EE, White BN. Differential permeability of rivers to raccoon gene flow corresponds to rabies incidence in Ontario, Canada. Mol Ecol. 2009; 18: 43–53. 10.1111/j.1365-294X.2008.03989.x 19140963

[136] Rees EE, Pond BA, Cullingham CI, Tinline R, Ball D, Kyle CJ, White BN. Assessing a landscape barrier using genetic simulation modelling: implications for raccoon rabies management. Prev Vet Med. 2008; 86: 107–123. 10.1016/j.prevetmed.2008.03.007 18440659

[137] Northrup JM, Hooten MB, Anderson CR, Wittemyer G. Practical guidance on characterizing availability in resource selection functions under a use-availability design. Ecology. 2013; 94: 1456–1463. 23951705 10.1890/12-1688.1

[138] Murray KA, Retallick RWR, Puschendorf R, Skerratt LF, Rosauer D, McCallum HI, et al Assessing spatial patterns of disease risk to biodiversity: implications for the management of the amphibian pathogen, *Batrachochytrium dendrobatidis*. J Appl Ecol. 2011; 48: 163–173.

[139] Ellis CK, Carroll DS, Lash RR, Townsend Peterson A, Damon IK, Malekani J, et al Ecology and geography of human monkeypox case occurrences across Africa. J Wildl Dis. 2012; 48: 335–347. 10.7589/0090-3558-48.2.335 22493109

[140] Yackulic CB, Chandler R, Zipkin EF, Royle JA, Nichols JD, Grant EHC, et al Presence-only modelling using MAXENT: when can we trust the inferences? Methods Ecol Evol. 2012; 4: 236–243.

[141] McRae BH, Dickson BG, Keitt TH, Shah VB. Using circuit theory to model connectivity in ecology, evolution, and conservation. Ecology. 2008; 89: 2712–2724. 18959309 10.1890/07-1861.1

[142] Algeo TP. Assessing raccoon (Procyon lotor) habitat use and predicting potential corridors for rabies spread with maximum entropy and circuit theory modeling. PhD Dissertation. University of New Hampshire. 2014.

[143] Shwiff S, Aenishaenslin C, Ludwig A, Berthiaume P, Bigras-Poulin M, Kirkpatrick K, et al Bioeconomic modelling of raccoon rabies spread management impacts in Quebec, Canada. Transbound Emerg Dis. 2013; 60: 330–337. 10.1111/j.1865-1682.2012.01351.x 22709550

[144] Chipman RB, Cozzens TW, Shwiff SA, Biswas R, Plumley J, O’Quin J, Algeo TP, Rupprecht CE, Slate D. Costs of raccoon rabies incidents in cattle herds in Hampshire County, West Virginia, and Guernsey County, Ohio. J Am Vet Med Assoc. 2013; 243: 1561–1567. 10.2460/javma.243.11.1561 24261805

[145] Kemere P, Liddel MK, Evanelou, P, et al. Economic analysis of a large scale oral vaccination program to control raccoon rabies. In: Clark L, Hone J, Shivik JA, et al., eds. Human Conflicts with wildlife: economic considerations. Fort Collins: USDA, APHIS, National Wildlife Research Center, 2002; 109–115.

[146] Elser JL, Bigler LL, Anderson AM, Maki JL, Lein DH, Shwiff SA. The economics of a successful raccoon rabies elimination program on Long Island, New York. PLoS Negl Trop Dis. 2016; 10: e0005062 10.1371/journal.pntd.0005062 27935946 PMC5147783

[147] Boulanger JR, Bigler LL, Curtis PD, Lein DH, Lembo AJ. A polyvinyl chloride bait station for dispensing rabies vaccine to raccoons in suburban landscapes. Wild Soc Bull. 2006; 34: 1206–1211.

[148] Algeo TP, Chipman RB, Bjorklund BM, Chandler MD, Wang X, Slate D, et al Anatomy of the Cape Cod oral rabies vaccination program In: Timm RM and Madon MB, editors. Proceedings of the 23^rd^ Vertebrate Pest Conference. Davis: The University of California; 2008 pp. 264–269.

[149] Singer A, Salman M, Thulke H-H. Reviewing model application to support animal health decision making. Prev Vet Med. 2011; 99: 60–67. 10.1016/j.prevetmed.2011.01.004 21306779

[150] MacInnes CD, Smith SM, Tinline RR, Ayers NR, Bachmann P, Ball DGA, et al Elimination of rabies from red foxes in eastern Ontario. J Wildl Dis. 2001; 37: 2001. 10.7589/0090-3558-37.1.119 11272485

[151] Rosatte RC, Power MJ, Donovan D, Davies JC, Allan M, Bachmann B, et al Elimination of arctic variant rabies in red foxes, metropolitan Toronto. Emerg Inf Dis. 2007c; 13: 25–27. 10.3201/eid1301.060622 PMC2725809 17370512

[152] Rosatte R, Allan M, Sobey K, Donovan D, Davies JC, Silver A, et al Prevalence of tetracycline and rabies virus antibody in raccoons, skunks, and foxes following aerial distribution of V-RG baits to control raccoon rabies in Ontario, Canada. J Wildl Dis. 2008; 44: 946–964. 10.7589/0090-3558-44.4.946 18957651

[153] Fearneyhough MG, Wilson PJ, Clark KA, Smith DR, Johnston DH, Hicks BN, et al Results of an oral rabies vaccination program for coyotes. J Am Vet Med Assoc. 1998; 212: 498–502. 9491156

[154] Horman JT, Shannon KV, Simpson EM, Burja TM, Fey RH, Smith JJ, et al Control of terrestrial animal rabies in Anne Arundel County, Maryland, after oral vaccination of raccoons (1998–2007). J Am Vet Med Assoc. 2012; 241: 725–734. 10.2460/javma.241.6.725 22947155

[155] Robbins AH, Borden MD, Windmiller BS, Niezgoda M, Marcus LC, O’Brien SM, et al Prevention of the spread of rabies to wildlife by oral vaccination of raccoons in Massachusetts. J Am Vet Med Assoc. 1998; 213: 1407–1412. 9828930

[156] Roscoe DE, Holste WC, Sorhage FE, Campbell C, Niezgoda M, Buchannan R, et al Efficacy of an oral vaccinia-rabies glycoprotein recombinant vaccine in controlling epidemic raccoon rabies in New Jersey. J Wildl Dis. 1998; 34: 752–763. 10.7589/0090-3558-34.4.752 9813845

[157] Sattler AC, Krogwold RA, Wittum TE, Rupprecht CE, Algeo TP, Slate D, et al Influence of oral rabies vaccine bait density on rabies seroprevalence in wild raccoons. Vaccine. 2009; 27: 7187–7193. 10.1016/j.vaccine.2009.09.035 19925951

[158] Bolzoni L, Dobson AP, Gatto M, De Leo GA. Allometric scaling and seasonality in the epidemics of wildlife diseases. Am Nat. 2008; 172: 818–826. 10.1086/593000 18947297

