# Correction: Management and modeling approaches for controlling raccoon rabies: The road to elimination

**DOI:** 10.1371/journal.pntd.0005579

**Published:** 2017-05-02

**Authors:** The PLOS Neglected Tropical Diseases Staff

The Fig 1 inset legend is incorrect. It contains a reference to Mongoose, which is neither a continental lineage, nor shown in the figure. The authors have provided a corrected version here.

**Fig 1:**
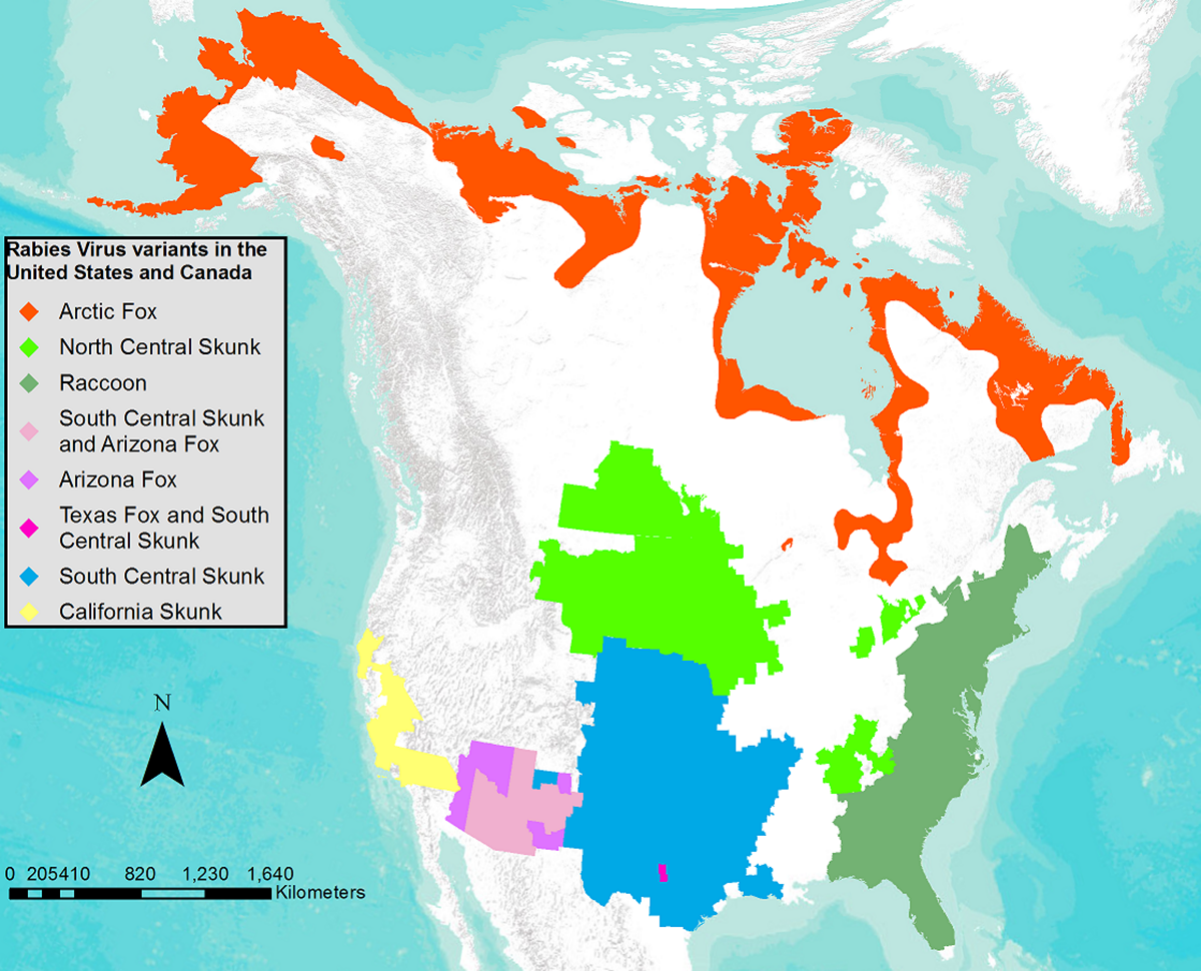
Current geographic distribution of rabies virus variants in the continental US and Canada.
